# nal‐IRI+5‐FU/LV versus 5‐FU/LV in post‐gemcitabine metastatic pancreatic cancer: Randomized phase 2 trial in Japanese patients

**DOI:** 10.1002/cam4.3558

**Published:** 2020-10-25

**Authors:** Makoto Ueno, Shoji Nakamori, Kazuya Sugimori, Masashi Kanai, Masafumi Ikeda, Masato Ozaka, Masayuki Furukawa, Takuji Okusaka, Ken Kawabe, Junji Furuse, Yoshito Komatsu, Hiroshi Ishii, Atsushi Sato, Satoshi Shimizu, Priti Chugh, Rui Tang, Tatsuya Ioka

**Affiliations:** ^1^ Department of Gastroenterology, Hepatobiliary and Pancreatic Medical Oncology Division Kanagawa Cancer Center Yokohama‐shi Kanagawa Japan; ^2^ Department of Surgery NHO Osaka National Hospital Osaka Japan; ^3^ Gastroenterological Center Yokohama City University Medical Center Yokohama‐shi Kanagawa Japan; ^4^ Department of Medical Oncology Kyoto University Hospital Kyoto Japan; ^5^ Department of Hepatobiliary and Pancreatic Oncology National Cancer Center Hospital East Chiba Japan; ^6^ Department of Gastroenterology Cancer Institute Hospital of JFCR Tokyo Japan; ^7^ Department of Hepatic‐biliary‐pancreatology NHO Kyushu Cancer Center Fukuoka Japan; ^8^ Department of Hepatobiliary and Pancreatic Oncology National Cancer Center Hospital Tokyo Japan; ^9^ Department of Medicine and Bioregulatory Sciences Graduate School of Medical Sciences Kyushu University Fukuoka Japan; ^10^ Faculty of Medicine Department of Medical Oncology Kyorin University Tokyo Japan; ^11^ Department of Cancer Chemotherapy Hokkaido University Hospital Cancer Center Hokkaido Japan; ^12^ Clinical Research Center Chiba Cancer Center Chiba Japan; ^13^ Department of Oncology Hirosaki University Hospital Aomori Japan; ^14^ Department of Gastroenterology Saitama Cancer Center Saitama Japan; ^15^ Servier Pharmaceuticals Boston MA USA; ^16^ Department of Cancer Survey and Gastrointestinal Oncology Osaka International Cancer Institute Osaka Japan

**Keywords:** chemotherapy, clinical trials, medical oncology, pancreatic cancer, pancreatic ductal adenocarcinoma

## Abstract

**Background:**

In the NAPOLI‐1 phase 3 trial, liposomal irinotecan (nal‐IRI) +5‐fluorouracil/leucovorin (5‐FU/LV) significantly increased mPFS versus 5‐FU/LV (3.1 vs. 1.5 months [unstratified HR = 0.56, *p* = 0.0001]) in patients with mPAC that progressed on prior gemcitabine‐based therapy. This randomized phase 2 trial evaluated nal‐IRI+5‐FU/LV tolerability (Part 1), safety, and efficacy (Part 2; outcomes reported here) in Japanese patients with mPAC that progressed on gemcitabine‐based therapy.

**Methods:**

Patients were randomized 1:1 and stratified by KPS (70 and 80 vs. ≥90) and baseline albumin (≥4.0 g/dl vs. <4.0 g/dl). Primary endpoint was PFS; secondary endpoints were ORR, DCR, OS, TTF, CA19‐9 response, and QoL. The ITT population comprised all randomized patients.

**Results:**

Patient characteristics differed between nal‐IRI+5‐FU/LV (n = 40) and 5‐FU/LV (n = 39) arms, including baseline hepatic lesions (63% vs. 51%), stage IV disease at diagnosis (78% vs. 51%), and post‐study anticancer therapy (55% vs. 72%). Investigator‐assessed mPFS increase with nal‐IRI+5‐FU/LV was clinically meaningful and statistically significant versus 5‐FU/LV (2.7 vs. 1.5 months, HR = 0.60). Independently assessed mPFS showed similar trends (1.7 vs. 1.6 months, HR = 0.79). mOS was 6.3 months with nal‐IRI+5‐FU/LV and not reached with 5‐FU/LV. ORR increased significantly with nal‐IRI+5‐FU/LV versus 5‐FU/LV (18% vs. 0, rate difference 17.5). Commonly reported grade ≥3 treatment‐emergent AEs were decreased neutrophil count (37% vs. 3%), decreased white blood cell count (20% vs. 0), and diarrhea (17% vs. 3%).

**Conclusions:**

In conclusion, clinically meaningful and statistically significant gains in investigator‐assessed PFS and ORR were observed with nal‐IRI+5‐FU/LV versus 5‐FU/LV in Japanese patients, with no new or unexpected safety signals. (Clinicaltrials.gov ID: NCT02697058).

## Graphical abstract

The phase 3 NAPOLI‐1 trial has previously shown the efficacy of liposomal irinotecan (nal‐IRI)+5‐FU/LV in treatment of patients with metastatic pancreatic cancer that has progressed on gemcitabine‐based therapy, but did not include any Japanese patients. This randomized phase 2 trial demonstrated clinically meaningful and statistically significant gains in investigator‐assessed progression‐free survival and objective response rate with nal‐IRI+5‐FU/LV vs 5‐FU/LV in Japanese patients. No novel or unexpected safety signals were observed in this population.

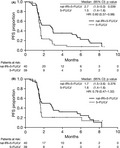

## 1 INTRODUCTION

Pancreatic cancer (PAC) is the sixth most common cancer and fourth‐leading cause of cancer‐related death in Japan, with over 43,000 new cases and 37,000 deaths in 2018.^1^ Additionally, PAC has recently been named the fourth‐leading cause of death due to cancer in the European Union and the United States. It is projected to become the second‐leading cause of cancer‐related death in the United States by 2030 and the third‐leading cause of cancer death in Europe, with a mortality rate approaching incidence.^2^, ^3^, ^4^, ^5^

The majority of patients (≥80%) are diagnosed with metastatic disease, making curative treatment virtually impossible and contributing to the observed mortality rates.^6^ Chemotherapy‐based, first‐line treatment options in patients with metastatic PAC (mPAC) and good Eastern Cooperative Oncology Group (ECOG) performance status (PS; 0–1) include gemcitabine+nab‐paclitaxel and the FOLFIRINOX (folinic acid, fluorouracil, irinotecan, oxaliplatin) regimen.^6^, ^7^, ^8^, ^9^ Patients with worse PS (≥2) may receive gemcitabine monotherapy, gemcitabine plus erlotinib, 5‐fluorouracil (5‐FU)‐based therapies including S‐1 (tegafur/gimeracil/oteracil), and best supportive care only.^6^, ^9^, ^10^, ^11^

Treatment options in patients with metastatic disease that progressed on gemcitabine‐based therapy include the recently approved combination regimen of liposomal irinotecan (nal‐IRI) plus 5‐FU/leucovorin (5‐FU/LV), and unapproved oxaliplatin‐based regimens (e.g., OFF [oxaliplatin, folinic acid, fluorouracil] or mFOLFOX6 [modified folinic acid, fluorouracil, oxaliplatin]).^12^, ^13^, ^14^, ^27^ Studies evaluating oxaliplatin‐based regimens have yielded conflicting results: CONKO‐003 reported increased overall survival (OS) with the OFF regimen, whereas PANCREOX (using mFOLFOX6) did not.^12^, ^13^

Recent clinical trials evaluated the use of S‐1 in combination with oxaliplatin, LV, or non‐liposomal irinotecan compared with S‐1 alone in Japanese patients with advanced or mPAC refractory to gemcitabine‐based therapy. The S‐1‐based combinations did not result in significant survival benefits in these patients compared with S‐1 monotherapy.^15^, ^16^, ^17^, ^18^

nal‐IRI consists of PEGylated liposomes containing irinotecan sucrosofate salt, a topoisomerase I inhibitor. Liposomal encapsulation reduces premature liver metabolism and conversion of irinotecan to the highly active SN‐38 metabolite. nal‐IRI exhibits a lower maximum concentration of free irinotecan in plasma, a longer half‐life and an increased area under the curve in plasma for SN‐38 compared with non‐liposomal irinotecan.^19^, ^20^, ^21^, ^22^, ^23^ This prolongs tumor exposure to SN‐38 above its antitumor activity threshold, and raises SN‐38 levels in tumor tissue compared with plasma.^19^, ^21^, ^23^

In the global NAPOLI‐1 phase 3 trial, nal‐IRI+5‐FU/LV significantly increased median OS versus 5‐FU/LV (median OS: 6.1 vs. 4.2 months; unstratified hazard ratio [HR] = 0.67; *p* = 0.012) in patients with metastatic pancreatic ductal adenocarcinoma that progressed after gemcitabine‐based therapy. Median investigator‐assessed progression‐free survival (PFS) was also improved in these patients (3.1 vs. 1.5 months; HR = 0.56; *p* = 0.0001). The regimen did not compromise quality of life (QoL), and resulted in increased quality‐adjusted time without symptoms of disease progression or grade ≥3 toxicity (QTWiST).^14^, ^24^, ^25^, ^26^, ^27^ The nal‐IRI+5‐FU/LV regimen is now included in treatment guidelines as a recommended and approved option for use in patients with mPAC that progressed after gemcitabine‐based therapy who have a suitable PS and comorbidity profile.^6^, ^10^, ^11^, ^28^ This study used the same nal‐IRI+5‐FU/LV dosing regimen to determine its efficacy and safety profile in Japanese patients.

## 2 MATERIALS AND METHODS

### 2.1 Study overview

This was a prospective, open‐label, randomized, multicenter phase 2 study in Japanese patients with mPAC that progressed or recurred following prior gemcitabine‐based therapy (registered at Clinicaltrials.gov, identifier: NCT02697058). Aspects of this work were previously presented at the ESMO Asia 2019 congress.^29^

The study consisted of two parts. Part 1 (safety run‐in) assessed the safety of nal‐IRI+5‐FU/LV to confirm the tolerability and to characterize the pharmacokinetics (PK) of the dosing regimen used in the NAPOLI‐1 trial. Patients in Part 1 continued in the study until occurrence of progressive disease (PD; radiologic or symptomatic deterioration) or unacceptable toxicity. After the Independent Data Monitoring Committee had reviewed all safety data, Part 2 was opened to further assess the safety of the combination, analyze the PK of nal‐IRI, and compare the efficacy of nal‐IRI+5‐FU/LV with 5‐FU/LV. In Part 2, patients were randomized 1:1 between the two treatment arms following stratification for Karnofsky Performance Status (KPS; 70 and 80 vs. ≥90) and baseline albumin levels (≥4.0 g/dl vs. <4.0 g/dl). Patients continued to receive treatment until PD (radiologic or symptomatic deterioration) or the occurrence of unacceptable toxicity.

The nal‐IRI+5‐FU/LV regimen consisted of 80 mg/m^2^ nal‐IRI (irinotecan hydrochloride trihydrate salt; equivalent to 70 mg/m^2^ irinotecan free base) administered by intravenous (IV) infusion over 90 min (±10 minutes). This was followed by 200 mg/m^2^ levoleucovorin calcium (LV) via IV infusion over 2 h, then, 2400 mg/m^2^ 5‐FU via IV infusion over 46 (±3) h, every 2 weeks (Q2W). The 5‐FU/LV regimen comprised 200 mg/m^2^ LV via IV infusion over 2 h, followed by 2400 mg/m^2^ 5‐FU via IV infusion over 46 (±3) h, Q2W. The 5‑FU/LV dose in both arms was based on the combination therapy dose of 5‐FU/LV in the NAPOLI‐1 trial.^14^, ^27^

During screening, all patients were assessed for eligibility and tested for the presence of *uridine*‐diphosphate glucuronosyl transferase 1A1 (UGT1A1)*28 and UGT1A1*6 alleles to determine the starting dose for nal‐IRI. A patient found to be homozygous or UGT1A1*28 or UGT1A1*6 or double heterozygous received a reduced starting dose for nal‐IRI (60 mg/m^2^ irinotecan hydrochloride trihydrate salt, equivalent to 50 mg/m^2^ irinotecan free base).

### 2.2 Inclusion and exclusion criteria

Patients had to be ≥20 years old, with histologically or cytologically confirmed adenocarcinoma of the exocrine pancreas and documented metastatic disease with ≥1 measurable lesion as defined by *Response Evaluation Criteria in Solid Tumors* (RECIST) v1.1 guidelines. Additional inclusion criteria were KPS ≥70, adequate bone marrow reserves, liver and renal function, and documented disease progression after prior gemcitabine or any gemcitabine‐containing therapy (excluding conventional irinotecan) in a locally advanced or metastatic setting. Prior chemotherapy must have been stopped for ≥21 days prior to first dose and patients had to have recovered from the effects of any prior surgery, radiotherapy, or other antineoplastic therapy with no residual adverse events (AEs) of grade ≥2.

Patients were excluded from the study if they had active and uncontrolled central nervous system (CNS) metastases; for controlled CNS metastases, patients were to have discontinued steroid medication for ≥28 days prior to starting study therapy. Further exclusion criteria included history of any secondary malignancy in the previous 5 years (patients with prior history of in situ cancer or basal or squamous cell skin cancers were eligible), severe arterial thromboembolic events (myocardial infarction, unstable angina pectoris, stroke) <6 months before inclusion, and significant cardiac conduction abnormalities including a history of long corrected QT interval by Frederica (QTcF) syndrome and/or pacemaker, New York Heart Association Class III or IV congestive heart failure, ventricular arrhythmias, or uncontrolled blood pressure. Patients were also ineligible if they were unable to discontinue potent human cytochrome P450 3A4 isoenzyme inducers within 2 weeks and inhibitors within 1 week before start of treatment, or had active infection or unexplained fever >38.5°C during screening visits or on the first scheduled day of dosing, which in the opinion of the investigator might have compromised the patient's participation in the study or influenced study outcome. In addition, known hypersensitivity to any of the components of nal‐IRI, other liposomal products, fluoropyrimidines, or LV was an exclusion criterion.

### 2.3 Efficacy outcomes (study Part 2)

The primary endpoint was PFS in Part 2, defined as the time from randomization to the first documented disease progression based on RECIST v1.1 or death due to any cause. Evaluation of disease progression was carried out by both independent and investigator assessments as per protocol. Secondary endpoints included objective response rate (ORR; the proportion of patients with a best overall response of unconfirmed complete response [CR] or partial response [PR]) and disease control rate (DCR; patients with a best overall response of unconfirmed CR, PR, or stable disease [SD] lasting ≥24 weeks following the first study drug administration). Response data were based on unconfirmed, investigator‐reported values.

OS was calculated as time from randomization to date of death due to any cause or the date of last known alive, and time to treatment failure (TTF) as time from randomization to disease progression according to RECIST v1.1, death due to any cause, discontinuation of treatment due to toxicity, symptomatic deterioration, or start of another anticancer therapy. Carbohydrate antigen 19‐9 (CA19‐9) tumor marker response was defined as a decrease of ≥50% versus baseline at least once during the treatment period. QoL was measured on a monthly basis, using the European Organization for Research and Treatment of Cancer Quality of Life Questionnaire C30 (EORTC‐QLQ‐C30).

### 2.4 Safety outcomes

Safety data were continuously collected from patient consent until 30 days after the last dose or before initiation of a new antineoplastic treatment. Safety measures included the incidence of serious AEs (SAEs), and incidence and intensity of nonserious AEs, which was coded to preferred term and system‐organ class using Medical Dictionary for Regulatory Activities (MedDRA), version 18.1 and graded according to the National Cancer Institute Common Terminology Criteria for Adverse Events (NCI‐CTCAE) version 4.03, as were laboratory tests. A treatment‐emergent AE (TEAE) was defined as an AE with an onset date or a preexisting AE worsening following the first dose of study drug until 30 days after the last dose of study drug. All TEAEs requiring dose reductions were considered treatment‐related.

### 2.5 Statistical analyses

The intention‐to‐treat (ITT) population was used for the primary analysis and included all patients randomized during study Part 2. All safety analyses were based on the safety population, comprising all patients receiving ≥1 dose of study drug. CA19‐9 tumor marker response and patient‐reported outcomes (PROs) were analyzed based on a subset of the primary efficacy analysis population evaluable for the outcome measure. PFS, OS, and TTF for each arm were estimated using the Kaplan–Meier method.

Primary efficacy outcome measures from the investigator's assessment were also evaluated. In case of a discrepancy between the assessment of the independent central review board and that of the investigator, the independent board's assessment of PFS took precedence. ORR, DCR, and CA19‐9 tumor marker response were compared using Fisher's exact test. HRs were based on unstratified Cox proportional hazards modeling. Two‐sided *p*‐values were determined using the log‐rank test. Statistical significance was defined at a level of *α* = 0.2. Assuming a dropout rate of 10%, approximately 74 patients were to be randomized. A total of 53 events (disease progression or death) would allow the study to have an approximately 88% power to detect a 1.5‐month improvement in PFS, based on a HR of 0.5. The total sample size was expected to be 80 patients, including the six patients enrolled in Part 1.

## 3 RESULTS

### 3.1 Patient baseline characteristics and demographics

**FIGURE 1:**
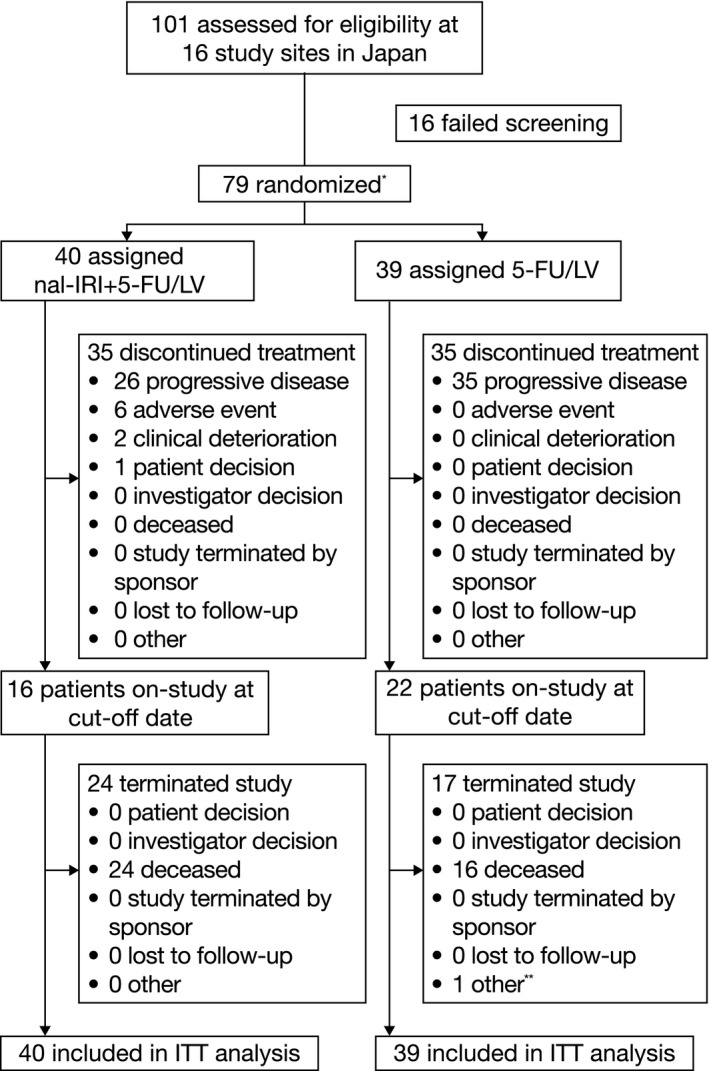
CONSORT diagram for patient disposition in study Part 2 (ITT population). *Not including patients receiving nal‐IRI+5‐FU/LV (n = 6) during study Part 1. The ITT population included all Part 2 patients even if they did not receive any study treatment. **One patient was randomized but not dosed as the patient was found to be in conflict with exclusion criteria prior to receiving study drug. 5‐FU, 5‐fluorouracil; ITT, intention‐to‐treat; LV, leucovorin; nal‐IRI, liposomal irinotecan

During the 22‐month study period from 30 March 2016 until 31 January 2018, 101 patients were assessed for eligibility at 16 participating Japanese centers (see Figure 1). Six patients were enrolled in study Part 1 and received nal‐IRI+5‐FU/LV, and following review of all safety data by the study's independent data monitoring committee, study Part 2 was opened. In Part 2, 79 patients were randomized to receive treatment with nal‐IRI+5‐FU/LV (n = 40) or 5‐FU/LV (n = 39). These patients were included in the ITT population. Patients (n = 6) from the safety run‐in of the nal‐IRI+5‐FU/LV regimen during study Part 1 were included in the safety population only.

Three patients (8%) in the nal‐IRI+5‐FU/LV arm and three patients (8%) in the 5‐FU/LV arm were homozygous for UGT1A1*28 or UGT1A1*6 or both; or heterozygous for both UGT1A*28 and UGT1A1*6. None of the patients in the nal‐IRI+5‐FU/LV arm presented with Gilbert syndrome or had clear suggestive symptoms of the disease in their medical history. Several differences in baseline characteristics were noted between the two treatment arms (Table 1). A lower proportion of patients receiving nal‐IRI+5‐FU/LV had a KPS score of 100 compared with patients receiving 5‐FU/LV (15% vs. 26%), whereas a greater proportion of patients in the nal‐IRI+5‐FU/LV arm had hepatic lesions at baseline (63% vs. 51%) and stage IV disease at diagnosis (78% vs. 51%). More patients in the 5‐FU/LV arm received ≥1 post‐study anticancer therapy than those receiving nal‐IRI+5‐FU/LV (72% vs. 55%).

**TABLE 1:** Patient characteristics and baseline demographics in study Part 2 (ITT population)

	nal‐IRI+5‐FU/LV n = 40	5‐FU/LV n = 39
Sex, n (%)		
Female	16 (40.0)	19 (48.7)
Male	24 (60.0)	20 (51.3)
Age, years		
Median	67.0	69.0
Min, max	39, 83	36, 78
Ethnicity, n (%)		
Asian (Japanese)	40 (100.0)	39 (100.0)
Baseline KPS, n (%)		
100	6 (15.0)	10 (25.6)
90	28 (70.0)	24 (61.5)
80	5 (12.5)	5 (12.8)
70	1 (2.5)	0
Baseline CA19‐9, n (%)^a^		
Median (IU/ml)	1419.4	1283.3
Evaluable for response	28 (70.0)	28 (71.8)
UGT1A1 status		
Homozygous for UGT1A1*28	0	1 (2.6)
Homozygous for UGT1A1*6	2 (5.0)	2 (5.1)
Heterozygous for both UGT1A1*28 and UGT1A1*6	1 (2.5)	0
Baseline albumin, n (%)		
<4.0 g/dl	31 (77.5)	30 (76.9)
≥4.0 g/dl	9 (22.5)	9 (23.1)
Median (g/dl)	37.0	38.0
Location of lesions at baseline, n (%)^b^		
Hepatic lobe	25 (62.5)	20 (51.3)
Lung	10 (25.0)	7 (17.9)
Pancreas	28 (70.0)	20 (51.3)
Para‐aortic lymph node	6 (15.0)	2 (5.1)
Supraclavicular lymph node	1 (2.5)	0
Other	23 (57.5)	26 (66.7)
Disease stage at diagnosis, n (%)		
IA	0	1 (2.6)
IB	0	0
IIA	1 (2.5)	2 (5.1)
IIB	0	5 (12.8)
III	8 (20.0)	11 (28.2)
IV	31 (77.5)	20 (51.3)
Primary tumor location, n (%)		
Head only	19 (47.5)	14 (35.9)
Body only	9 (22.5)	12 (30.8)
Tail only	10 (25.0)	9 (23.1)
Multi‐locations incl. head	0 (0.0)	0 (0.0)
Multi‐locations excl. head	2 (5.0)	2 (5.1)
Unknown	0	2 (5.1)
Previous lines of metastatic therapy, n (%)		
≤1	34 (85.0)	28 (71.8)
≥2	6 (15.0)	10 (25.6)
Prior anticancer therapy, n (%)^c^		
Gemcitabine‐containing	40 (100.0)	38 (97.4)^d^
Fluorouracil‐containing (S−1)	8 (20.0)	14 (35.9)
Fluorouracil‐containing (5‐FU)	0	1 (2.6)
Platinum‐containing (oxaliplatin)	1 (2.5)	0
Irinotecan‐containing	0	0
Investigational agents	0	3 (7.7)
Other	38 (95.0)	34 (87.2)
Administration setting of prior anticancer therapy, n (%)		
Neoadjuvant	1 (2.5)	4 (10.3)
Adjuvant	5 (12.5)	14 (35.9)
Advanced/metastatic	38 (95.0)	37 (94.9)
Consolidation	1 (2.5)	0
Unknown	0	0
Median time since last prior anticancer therapy, months (Min–Max)	0.9 (0.6–1.8)	0.9 (0.7–1.4)
Post‐study anticancer therapy, n (%)^c^		
Received ≥1 post‐study anticancer therapy	22 (55.0)	28 (71.8)
Gemcitabine‐containing	6 (15.0)	9 (23.1)
Fluorouracil‐containing (S−1)	12 (30.0)	7 (17.9)
Fluorouracil‐containing (5‐FU)	15 (37.5)	23 (59.0)
Platinum‐containing (oxaliplatin)	13 (32.5)	25 (64.1)
Irinotecan‐containing	9 (22.5)	19 (48.7)
Investigational agents	0	0
Other	4 (10.0)	7 (17.9)
Not recorded	18 (45.0)	11 (28.2)
Median time from last study drug exposure to first post‐study anticancer therapy, weeks (1st and 3rd quartiles)	3.1 (2.9, 4.7)	2.6 (2.1, 3.1)
Median time since initial diagnosis, months (1st and 3rd quartiles)	7.2 (4.2, 14.0)	10.8 (6.9, 20.8)

Notes

Baseline was defined as the last non‐missing value prior to first dose of study drug (i.e., nal‐IRI for nal‐IRI+5‐FU/LV arm or 5‐FU for 5‐FU/LV arm).

Baseline KPS and baseline albumin are the values defined at screening.

Time since last anticancer therapy was defined as the time between the most recent regimen date and the date of first dose of study drug (Part 1) or date of randomization (Part 2).

Abbreviations: 5‐FU, 5‐fluorouracil; CA19‐9, carbohydrate antigen 19‐9; KPS, Karnofsky performance status; LV, leucovorin; nal‐IRI, liposomal irinotecan; S‐1, tegafur/gimeracil/oteracil.

^a^ Only patients with a recorded pretreatment CA19‐9 value were included.

^b^ Lesion locations were defined according to *Response Evaluation Criteria In Solid Tumors* (RECIST) guidelines v1.1.

^c^ Column values may add up to ≥100% as patients could have received more than one prior line of therapy or more than one post‐study anticancer therapy, resulting in their inclusion in multiple categories.

^d^ One patient randomized to the 5‐FU/LV arm was found not to have received prior gemcitabine following randomization and did not receive study treatment.

### 3.2 Efficacy results

#### 3.2.1 Primary endpoint

**FIGURE 2:**
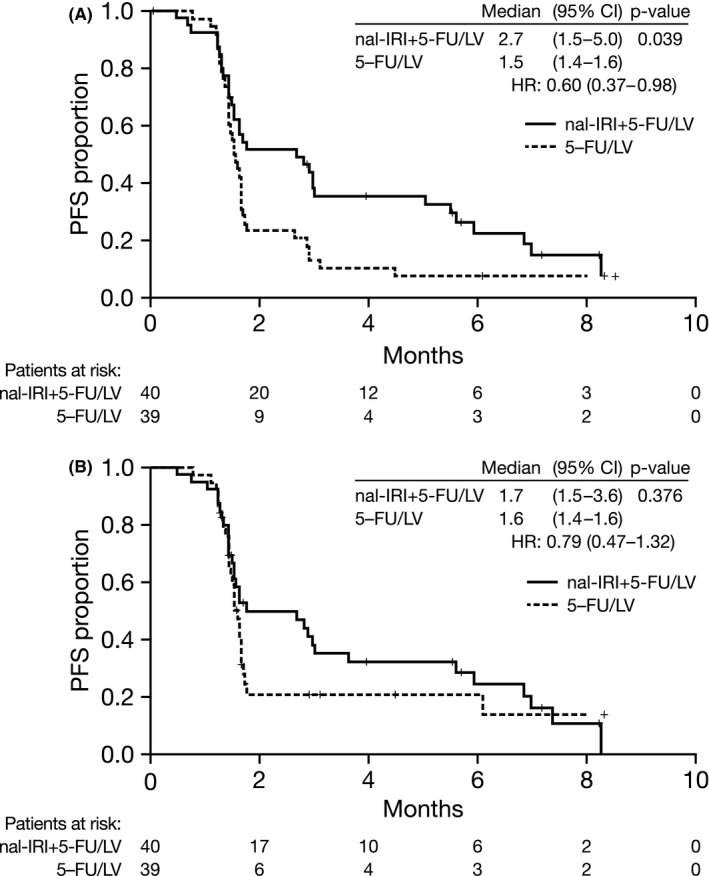
Progression‐free survival in study Part 2 (ITT population). Kaplan–Meier plots for PFS based on investigator assessment (A) and independent assessment (B) in the Part 2 ITT population. Tick marks indicate censoring points. 5‐FU, 5‐fluorouracil; CI, confidence interval; HR, hazard ratio; ITT, intention‐to‐treat; LV, leucovorin; nal‐IRI, liposomal irinotecan; PFS, progression‐free survival

At the primary analysis cutoff (4 May 2017), patients in the nal‐IRI+5‐FU/LV treatment arm achieved a median PFS by investigator assessment of 2.7 months (95% confidence interval [CI]: 1.5–5.0) compared with 1.5 months (95% CI: 1.4–1.6) in the control 5‐FU/LV arm (HR=0.60, *p* = 0.039; see Figure 2A and Table 2). According to independent assessment, patients in the nal‐IRI+5‐FU/LV arm achieved a median PFS of 1.7 months (95% CI: 1.5–3.6) with nal‐IRI+5‐FU/LV and 1.6 months (1.4–1.6) with 5‐FU/LV (HR = 0.79, *p* = 0.376; see Figure 2B and Table 2). As KPS and baseline albumin levels were used as stratification factors during randomization, a stratified analysis for PFS based on independent assessment was performed for the ITT analysis set using these stratification factors. These results were aligned with the primary analysis, with median PFS of 1.7 versus 1.6 months (HR = 0.76, 95% CI 0.44–1.29). A multivariate analysis of the ITT population indicated that treatment (HR = 0.65, *p* = 0.111) and presence of liver metastases (HR = 2.83, *p* = 0.001) were important factors affecting disease progression. Results from a sensitivity analysis excluding the six patients who were heterozygous or homozygous for UGT1A1*6 or UGT1A1*28 mutations showed no significant change in independently assessed PFS outcomes in either study arm (nal‐IRI+5‐FU/LV vs. 5‐FU/LV: 1.7 vs. 1.5 months, HR 0.68 [95% CI 0.40–1.19], *p* = 0.177).

**TABLE 2:** Summary of efficacy data for study Part 2 (primary analysis, ITT population)

	nal‐IRI+5‐FU/LV n = 40	5‐FU/LV n = 39
Progression‐free survival time (investigator assessment), months		
Median	2.7	1.5
95% CI	1.5–5.0	1.4–1.6
HR^a^	0.60	
95% CI	0.37–0.98	
*p*‐value^b^	0.039	
Progression‐free survival time (independent assessment), months		
Median	1.7	1.6
95% CI	1.5–3.6	1.4–1.6
HR^a^	0.79	
95% CI	(0.47–1.32)	
*p*‐value^b^	0.376	
Overall survival time, months		
Median	6.3	NR
95% CI	5.2–NR	6.1–NR
HR^a^	1.67	
95% CI	0.88–3.16	
*p*‐value^b^	0.110	
Time to treatment failure, months		
Median	1.7	1.5
95% CI	1.5–2.2	1.4–1.6
HR^a^	0.70	
95% CI	0.44–1.12	
*p*‐value^b^	0.134	
Best overall response,^c^ n (%)	40 (100.0)	39 (100.0)
ORR	7 (17.5)	0 (0)
Rate difference (95% CI), *p*‐value^d^	17.5 (5.7–29.3), 0.012	
CR	2 (5.0)	0
PR	5 (12.5)	0
SD	14 (35.0)	10 (25.6)
PD	19 (47.5)	27 (69.2)
Non‐CR/non‐PD	0	0
NE	0	2 (5.1)
Disease control rate,^e^ n (%)	8 (20.0)	2 (5.1)
Rate difference (95% CI), *p*‐value^d^	14.9 (0.7–29.1), 0.087	
Tumor marker CA19‐9 response, TMRE population^f^		
CA19‐9 response rate,^g^ n/N (%)	5/28 (17.9)	1/28 (3.6)
Rate difference (95% CI), *p*‐value^d^	14.3 (−1.5–30.1), 0.193	

Abbreviations: 5‐FU, 5‐fluorouracil; CA19‐9, carbohydrate antigen 19‐9, CI, confidence interval; CR, complete response; HR, hazard ratio; LV, leucovorin; nal‐IRI, liposomal irinotecan; NE, not evaluable; NR, not reached; ORR, objective response rate; PR, partial response; SD, stable disease; TMRE population, tumor marker response evaluable population.

^a^ HR was computed from unstratified Cox proportional hazards modeling.

^b^ Two‐sided *p*‐value from log‐rank test.

^c^ Best overall response was defined according to RECIST v1.1 and reviewed by an independent central review board. Tumor responses were measured and recorded every 6 weeks ±1 week.

^d^ Two‐sided *p*‐value from Fisher's exact test.

^e^ Disease control was defined as subjects with a best overall response of unconfirmed complete response, partial response, or stable disease lasting ≥24 weeks following the start of first study drug.

^f^ The TMRE population comprised patients who had elevated CA19‐9 levels (>30 IU/ml) at baseline and ≥1 post‐baseline CA19‐9 assessment.

^g^ CA19‐9 response was assessed through measurement of CA19‐9 serum levels within 7 days before the start of treatment (baseline) and subsequently every 6 weeks after enrollment, consistent with the tumor assessment schedule.

#### 3.2.2 Secondary endpoints

The independently assessed ORR with nal‐IRI+5‐FU/LV was 18% versus 0 with 5‐FU/LV (rate difference 17.5, *p* = 0.012) and disease control rates were 20% versus 5% (rate difference 14.9, *p* = 0.087, Table 2). A larger proportion of patients receiving nal‐IRI+5‐FU/LV experienced CR, PR, or SD compared with those treated with 5‐FU/LV, while fewer patients in the nal‐IRI+5‐FU/LV arm had PD (Table 2). At primary analysis cutoff, 60% of OS events had occurred in the nal‐IRI+5‐FU/LV arm compared with 41% in the 5‐FU/LV arm. Median OS was 6.3 months in the na‐IRI+5‐FU/LV treatment arm, but was not reached in the 5‐FU/LV control arm for this primary analysis (HR 1.67; Table 2 and Figure S1).

The greater proportion of patients in the control arm receiving post‐study therapy may be a confounding factor in the OS analysis. In the nal‐IRI+5‐FU/LV arm, 25% patients (n = 10) received a gemcitabine‐based or FOLFIRINOX regimen, versus 59% (n = 23) in the 5‐FU/LV arm. Therefore, a post hoc sensitivity analysis was carried out in which survival time was censored at the start date of FOLFIRINOX or gemcitabine‐based therapies. The median OS values from this analysis were 6.2 versus 6.7 months, respectively (HR = 1.11, 95% CI 0.49–2.49) (Table S1).

**FIGURE 3:**
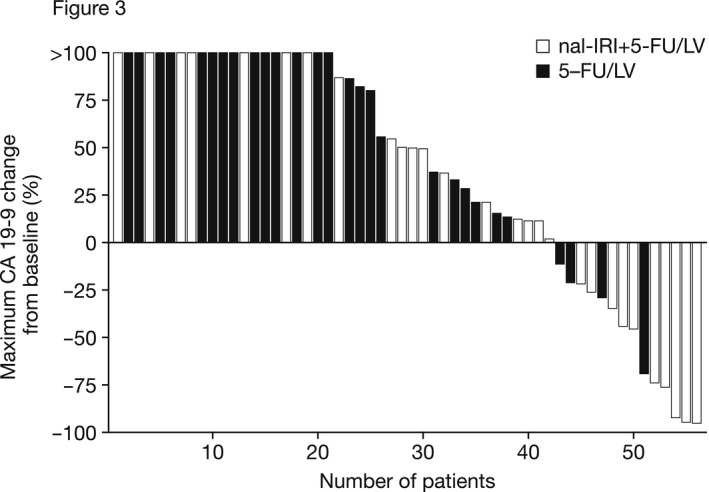
Change from baseline in CA19‐9 tumor marker response in study Part 2 (TMRE population). 5‐FU, 5‐fluorouracil; CA19‐9, carbohydrate antigen 19‐9; LV, leucovorin; nal‐IRI, liposomal irinotecan; TMRE, tumor marker response evaluable

TTF was comparable between the nal‐IRI+5‐FU/LV and 5‐FU/LV arms (1.7 months vs. 1.5 months, HR = 0.70; see Table 2). The tumor marker response evaluable (TMRE) population comprised 28 patients each in each arm. More patients treated with nal‐IRI+5‐FU/LV achieved a CA19‐9 response compared with those receiving 5‐FU/LV (18% vs. 4%; see Figure 3 and Table 2), although this difference was not statistically significant (*p* = 0.193).

Baseline EORTC‐QLQ‐C30 scores were similar between the nal‐IRI+5‐FU/LV and 5‐FU/LV treatment arms apart from minor differences in global health status (median score 75 vs. 58), and the cognitive (100 vs. 83), emotional (83 vs. 92), social (100 vs. 83), as well as pain (17 vs. 33) functioning and symptom scales (Table S2). Both treatment arms had baseline values in the high range (>75) for physical, role, cognitive, emotional, and social functioning scales (Table S2). Differences between treatment arms regarding change of median score from baseline until week 12 were limited, occurring in the global health status (0 vs. 8), role functioning (0 vs. 33), and fatigue symptom scale (0 vs. −11) scores (Table S2).

### 3.3 Safety and tolerability results

#### 3.3.1 Treatment duration and dose intensity

Fewer patients receiving nal‐IRI+5‐FU/LV had a minimum time on treatment of ≥6 weeks (76%) compared with those treated with 5‐FU/LV (84%; Table 3). However, the proportion of patients receiving treatment for ≥12 or ≥18 weeks in the nal‐IRI+5‐FU/LV arm was approximately twofold higher than in the 5‐FU/LV arm.

**TABLE 3:** Treatment duration and exposure in the nal‐IRI+5‐FU/LV and 5‐FU/LV treatment arms (safety population)

	nal‐IRI+ 5‐FU/LV n = 46	5‐FU/LV n = 38
Number of treatment cycles received		
Mean (SD)	6.7 (5.7)	4.9 (4.1)
Median (1st and 3rd quartiles)	3.5 (3.0, 11.0)	3.5 (3.0, 4.0)
Minimum time on treatment, n (%)		
≥6 weeks	35 (76.1)	32 (84.2)
≥12 weeks	19 (41.3)	9 (23.7)
≥18 weeks	15 (32.6)	4 (10.5)
Relative dose intensity (%), mean (SD)^a^		
nal‐IRI	91.5 (13.3)	n/a
5‐FU	90.7 (12.4)	99.0 (4.4)
Duration of exposure (weeks), mean (SD)^b^		
nal‐IRI	15.1 (12.6)	n/a
5‐FU	15.0 (12.7)	10.2 (8.5)

Note

The safety population comprised patients who received ≥1 dose of study drug during study Part 1 or 2. Treatment cycles were 2 weeks in both arms.

Abbreviations: 5‐FU, 5‐fluorouracil; LV, leucovorin; nal‐IRI, liposomal irinotecan; n/a, not applicable; SD, standard deviation.

^a^ Relative dose intensity = (actual dose intensity/planned dose intensity) ×100.

^b^ Duration of exposure is the total duration (start date of last dose – start date of first dose +14)/7.

#### 3.3.2 Treatment‐emergent adverse events

Overall, TEAEs were in the “Blood and lymphatic system disorders” or “gastrointestinal disorders” system‐organ classes and were more frequent in patients receiving nal‐IRI+5‐FU/LV than in those who were administered 5‐FU/LV; grade ≥3 TEAEs occurred more frequently in 78% versus 37% patients (Table 4). In the nal‐IRI+5‐FU/LV arm, 61% of grade ≥3 TEAEs were judged as treatment‐related, versus 21% in the 5‐FU/LV arm, with decreased neutrophil count (37%), decreased white blood cell count (20%), and diarrhea (17%) being most commonly reported in the combination arm. The most frequently reported grade ≥3 TEAEs among patients in the control arm were decreased lymphocyte count (8%) and tumor pain (5%). Neutropenia of any grade occurred in 11% of patients receiving nal‐IRI+5‐FU/LV and none receiving 5‐FU/LV. There was one case of febrile neutropenia (2%) in the nal‐IRI+5‐FU/LV arm. Grade 3 and grade 4 treatment‐related TEAEs were recorded in patients with mutations in *UGT1A1* (homozygous UGT1A1*28, homozygous UGTA1A1*6, or heterozygous UGT1A1*28/*6; n = 3 in each arm). All three patients in the nal‐IRI+5‐FU/LV arm had grade ≥3 neutrophil count decreased, with one patient developing grade ≥3 neutropenia; none of the three patients developed grade ≥3 diarrhea.

**TABLE 4:** Grade ≥3 TEAEs in any treatment arm (safety population)

n (%)	nal‐IRI+5‐FU/LV n = 46	5‐FU/LV n = 38
Any grade ≥3 TEAE	36 (78.3)	14 (36.8)
Neutrophil count decreased	17 (37.0)	1 (2.6)
White blood cell count decreased	9 (19.6)	0
Diarrhea	8 (17.4)	1 (2.6)
Hyponatremia	4 (8.7)	0 (0.0)
Neutropenia	4 (8.7)	0 (0.0)
Anemia	3 (6.5)	1 (2.6)
Cholangitis	3 (6.5)	0
Gamma‐glutamyl transferase increased	3 (6.5)	0
Hypokalemia	3 (6.5)	0
Lymphocyte count decreased	1 (2.2)	3 (7.9)
Tumor pain	0	2 (5.3)
Decreased appetite	0	1 (2.6)
Fatigue	1 (2.2)	1 (2.6)
Febrile neutropenia	1 (2.2)	0
Nausea	1 (2.2)	1 (2.6)

Note

The safety population comprised patients who received ≥1 dose of study drug during study Part 1 or 2. TEAE was defined as AE with an onset date or a preexisting AE worsening following the first dose of study drug through to 30 days after the last dose of study drug. AEs were coded according to MedDRA version 18.1.

Abbreviations: 5‐FU, 5‐fluorouracil; AE, adverse event; LV, leucovorin; nal‐IRI, liposomal irinotecan; TEAE, treatment‐emergent adverse event.

#### 3.3.3 TEAEs of any grade leading to dose modification, reduction, delay, or treatment discontinuation

TEAEs of any grade leading to dose modifications were more frequent in patients in the nal‐IRI+5‐FU/LV arm than in those in the 5‐FU/LV arm (76% vs. 32%; see Table S3). Of these, 72% versus 16% were considered treatment‐related. A larger proportion of patients treated with nal‐IRI+5‐FU/LV required dose delays, reductions, or treatment discontinuation compared with patients receiving 5‐FU/LV.

In patients receiving nal‐IRI+5‐FU/LV, decreased white blood cell count (46%), decreased neutrophil count (44%), and diarrhea (11%) were the most frequent TEAEs leading to dose delay. Decreased neutrophil count (24%), diarrhea (17%), and decreased white blood cell count (15%) were also commonly reported as leading to dose reduction. The most common TEAEs leading to dose delay with 5‐FU/LV were decreased white blood cell count, constipation, and decreased platelet count (all 5%). Ten patients in the nal‐IRI+5‐FU/LV arm (22%) discontinued treatment during the study, with five (11%) deemed treatment‐related. Disease progression was responsible for two withdrawals.

#### 3.3.4 TEAEs leading to death

None of the TEAEs with fatal outcome in either arm was judged as treatment‐related. In patients receiving nal‐IRI+5‐FU/LV, four (9%) TEAEs with fatal outcome were reported: one due to infection (2%) and three due to progression of pancreatic carcinoma (7%; see Table S3). Two patients (5%) in the 5‐FU/LV arm died, both due to pancreatic carcinoma progression.

## 4 DISCUSSION

As previously discussed, in the NAPOLI‐1 phase 3 study, nal‐IRI+5‐FU/LV significantly increased PFS and OS compared with 5‐FU/LV in patients with mPAC that progressed on prior gemcitabine‐based therapy.^27^ In the current study, independently assessed median PFS was slightly longer with nal‐IRI+5‐FU/LV versus 5‐FU/LV (1.7 vs. 1.6 months). Importantly, investigator‐assessed median PFS was significantly increased with nal‐IRI+5‐FU/LV compared with 5‐FU/LV. This is consistent with results from the NAPOLI‐1 study, which also reported median PFS based on investigator assessment.^27^ It should be noted that tumor assessments were scheduled at discrete 6‐week intervals (±1 week) during the current study, increasing the likelihood of numerically similar estimates. nal‐IRI+5‐FU/LV increased ORR, DCR, TTF, and CA19‐9 response versus 5‐FU/LV, with ORR reaching statistical significance.

Median OS with nal‐IRI+5‐FU/LV compared with 5‐FU/LV could not be determined, as this was not reached in the 5‐FU/LV arm by the time of the primary analysis. However, the observed median OS in the nal‐IRI+5‐FU/LV arm is in line with expectations and prior findings from the NAPOLI‐1 study.^27^ A final analysis for OS conducted after the final data cutoff determined the median OS as 6.3 months in the nal‐IRI+5‐FU/LV arm and 9.1 months in the 5‐FU/LV arm (HR = 1.24, *p* = 0.371). The median OS among patients in the 5‐FU/LV arm exceeded the median OS of 4.2 months previously observed in the NAPOLI‐1 study.^27^

It should be noted that the primary endpoint of this study was PFS in study Part 2, while OS was a secondary endpoint. Unlike the NAPOLI‐1 trial, this study was not designed to detect a difference in OS between the treatment arms, and consequently has a sample size insufficient to draw robust conclusions in light of this finding.

Moreover, a smaller proportion of patients in the nal‐IRI+5‐FU/LV arm received post‐study anticancer therapy than in the 5‐FU/LV arm (55% vs. 72%). Further to this finding, a post hoc analysis conducted following the final data cutoff showed a greater proportion of patients in the 5‐FU/LV arm were subsequently treated with either a gemcitabine‐ (28%), oxaliplatin‐ (67%), or irinotecan‐based (51%) regimen compared with patients in the nal‐IRI+5‐FU/LV arm (20%, 48%, or 23%), consistent with observations from the primary analysis. This is in contrast to the NAPOLI‐1 study, where post‐progression therapy was similar between treatment arms.^27^

Gemcitabine‐, oxaliplatin‐, and irinotecan‐based combinations (i.e., gemcitabine+nab‐paclitaxel and FOLFIRINOX) are considered as front‐line therapy for patients with mPAC by current treatment guidelines.^6^, ^9^, ^10^, ^11^ It may be anticipated that those patients sufficiently fit to tolerate these regimens following termination of study treatment were more likely to be selected and could have prolonged survival compared with those unable to receive further treatment. The proportion of patients with a minimum time on treatment of ≥12 or ≥18 weeks was markedly lower in the 5‐FU/LV arm compared with the nal‐IRI+5‐FU/LV arm. We hypothesize that patients in the 5‐FU/LV arm likely experienced earlier disease progression than those in the nal‐IRI+5‐FU/LV arm and proceeded to efficacious post‐study anticancer therapy sooner, thereby increasing their survival time. These imbalances in post‐study anticancer therapy likely seriously confound the median OS determined in the primary analysis.

Additionally, as platinum‐based therapies are known to be particularly efficacious for patients with homologous recombination deficient (HRD) pancreatic cancer,^30^ there may be a subset of patients with HRD tumors in the 5‐FU/LV arm who derived a greater benefit from oxaliplatin‐based therapies. It is important to note that HRD status was not collected for patients in this study, so any direct effect of HRD cannot be analyzed.

In terms of number of prior lines of therapy, a lower proportion of patients in the NAPOLI‐1 trial had received 0–1 prior lines of metastatic therapy compared with the present study (66% vs. 85% for nal‐IRI+5‐FU/LV; 69% vs. 71.8% for 5‐FU/LV).^27^ A subanalysis of the NAPOLI‐1 trial indicated that patients who had received 0–1 prior lines of metastatic therapy had better PFS outcomes when receiving nal‐IRI+5‐FU/LV versus 5‐FU/LV, suggesting that earlier use of nal‐IRI+5‐FU/LV provides better PFS outcomes for patients.^31^ However, it is unclear whether the higher proportion of patients with 0–1 prior lines of therapy in the present study influenced outcomes, as investigator‐assessed PFS is similar in NAPOLI‐1 and the present study for both arms. In terms of types of prior treatment, only one patient in the nal‐IRI+5‐5‐FU/LV arm received a platinum‐containing therapy prior to the study, whereas in NAPOLI‐1 32% patients in the nal‐IRI+5‐FU/LV arm and 34% patients in the 5‐FU/LV arm had received a platinum‐based prior therapy.^27^ It is unclear how these differences in prior treatment types between the two trials may have affected outcomes in the Japanese study population. It is important to note that NAPOLI‐1 was a much larger, international trial, so patients had more opportunities to receive a wider range of treatments than in the present study.^27^

In addition, differences in patient baseline characteristics between the two treatment arms may also have contributed to the observed OS outcome. For example, patients in the nal‐IRI+5‐FU/LV arm had, on average, an increased incidence of presence of hepatic lesions, and a lower KPS. It is also important to note that a higher proportion of patients in the nal‐IRI+5‐FU/LV arm had stage IV disease at diagnosis versus patients in the 5‐FU/LV arm (77.5% vs. 51.3%). This may have resulted in better survival outcomes for patients in the 5‐FU/LV arm; this possibility is supported by data from a recent subanalysis of NAPOLI‐1 showing that patients with stage III disease at diagnosis had better OS outcomes than patients with stage IV disease in both the overall ITT population and in both treatment arms.^31^

QoL was maintained throughout the course of treatment despite the late‐stage disease and the addition of nal‐IRI to the 5‐FU/LV regimen. This is consistent with findings from a QoL analysis of patients receiving the nal‐IRI+5‐FU/LV regimen in the NAPOLI‐1 trial.^25^ The most frequently reported grade 3/4 TEAEs were typically myelosuppressive and gastrointestinal.

The incidence of all‐grade neutropenia with nal‐IRI+5‐FU/LV was 11% (8% grade ≥3), with one case of febrile neutropenia; the incidence of diarrhea was comparable to that previously reported for patients receiving the regimen in the NAPOLI‐1 trial.^27^ Decreased white blood cell and neutrophil counts, as well as diarrhea, were among the TEAEs most commonly leading to dose delays or reductions in patients treated with nal‐IRI+5‐FU/LV, though seldom leading to treatment discontinuation. Of the TEAEs with fatal outcome in either treatment arm, none was judged as treatment‐related, and all but one were associated with the underlying disease. There was a higher instance of neutropenia in patients receiving nal‐IRI+5‐FU/LV who had mutations in *UGT1A1* (homozygous for UGT1A1*6 [n =2] or heterozygous for UGT1A1*6 and UGT1A1*28 [n =1]). Although no firm conclusions can be drawn regarding safety in this small patient population, it is important to monitor patients with known *UGT1A1* mutations for neutropenia.

In conclusion, no new, unique, or unexpected safety signals were identified among Japanese patients with mPAC that progressed or recurred following prior gemcitabine‐based therapy. The safety profile of nal‐IRI+5‐FU/LV observed in this study was consistent with the global NAPOLI‐1 study and other clinical studies evaluating the regimen in this setting.^19^, ^23^, ^27^, ^32^ Additionally, clinically meaningful and statistically significant gains in investigator‐assessed PFS and ORR were observed with nal‐IRI+5‐FU/LV versus 5‐FU/LV. The data presented here are mostly comparable to prior experience with nal‐IRI+5‐FU/LV in the global NAPOLI‐1 trial and support the use of the regimen in a Japanese patient population.

## ETHICAL DISCLOSURE

The study protocol, informed consent form, and all amendments were reviewed and approved by an independent ethics committee prior to implementation. This study was conducted in accordance with the Declaration of Helsinki, the International Council for Harmonization Guideline for Good Clinical Practice E6 (April 1996), Title 21 of the US Code of Federal Regulations, the European Clinical Trial Directive (2001/20/EC and 2005/28/EC), and applicable national and local regulatory requirements (Ministry of Health, Labor and Welfare [MHLW] Ordinance No. 28, 27 March 1997, partially revised by MHLW Ordinance and their related notifications).

## AUTHOR CONTRIBUTIONS

Makoto Ueno: Conceived and designed the article, contributed and managed study material or patients, collected and assembled data, analyzed and interpreted data, contributed to writing the manuscript, approved the final version of the manuscript. Shoji Nakamori: Contributed and managed study material or patients, collected and assembled data, analyzed and interpreted data, contributed to writing the manuscript, approved the final version of the manuscript. Kazuya Sugimori: Contributed and managed study material or patients, collected and assembled data, analyzed and interpreted data, contributed to writing the manuscript, approved the final version of the manuscript. Masashi Kanai: Contributed and managed study material or patients, collected and assembled data, analyzed and interpreted data, contributed to writing the manuscript, approved the final version of the manuscript. Masafumi Ikeda: Contributed and managed study material or patients, collected and assembled data, analyzed and interpreted data, contributed to writing the manuscript, approved the final version of the manuscript. Masato Ozaka: Contributed and managed study material or patients, collected and assembled data, analyzed and interpreted data, contributed to writing the manuscript, approved the final version of the manuscript. Masayuki Furukawa: Contributed and managed study material or patients, collected and assembled data, analyzed and interpreted data, contributed to writing the manuscript, approved the final version of the manuscript. Takuji Okusaka: Contributed and managed study material or patients, collected and assembled data, analyzed and interpreted data, contributed to writing the manuscript, approved the final version of the manuscript. Ken Kawabe: Contributed and managed study material or patients, collected and assembled data, analyzed and interpreted data, contributed to writing the manuscript, approved the final version of the manuscript. Junji Furuse: Contributed and managed study material or patients, collected and assembled data, analyzed and interpreted data, contributed to writing the manuscript, approved the final version of the manuscript. Hiroshi Ishii: Contributed and managed study material or patients, collected and assembled data, analyzed and interpreted data, contributed to writing the manuscript, approved the final version of the manuscript. Yoshito Komatsu: Contributed and managed study material or patients, collected and assembled data, analyzed and interpreted data, contributed to writing the manuscript, approved the final version of the manuscript. Atsushi Sato: Contributed and managed study material or patients, collected and assembled data, analyzed and interpreted data, contributed to writing the manuscript, approved the final version of the manuscript. Satoshi Shimizu: Contributed and managed study material or patients, collected and assembled data, analyzed and interpreted data, contributed to writing the manuscript, approved the final version of the manuscript. Priti Chugh: Collected and assembled data, analyzed and interpreted data, contributed to writing the manuscript, approved the final version of the manuscript. Rui Tang: Collected and assembled data, analyzed and interpreted data, contributed to writing the manuscript, approved the final version of the manuscript. Tatsuya Ioka: Conceived and designed the article, contributed and managed study material or patients, collected and assembled data, analyzed and interpreted data, contributed to writing the manuscript, approved the final version of the manuscript.

## Supporting information

Fig S1

Table S1

Table S2

Table S3

## Data Availability

Individual participant data, including data dictionaries, will not be available for this study.

## References

[1] Ferlay J , Ervik M , Lam F , et al. Global cancer observatory: cancer today. Lyon, France: International Agency for Research on Cancer; 2018 https://gco.iarc.fr/today

[2] Ferlay J , Colombet M , Soerjomataram I , et al. Cancer incidence and mortality patterns in Europe: estimates for 40 countries and 25 major cancers in 2018. Eur J Cancer. 2018;103:356–387. 30100160 10.1016/j.ejca.2018.07.005

[3] Siegel RL , Miller KD , Jemal A . Cancer statistics, 2019. CA Cancer J Clin. 2019;69:7–34. 30620402 10.3322/caac.21551

[4] Rahib L , Smith BD , Aizenberg R , Rosenzweig AB , Fleshman JM , Matrisian LM . Projecting cancer incidence and deaths to 2030: the unexpected burden of thyroid, liver, and pancreas cancers in the United States. Cancer Res. 2014;74:2913–2921. 24840647 10.1158/0008-5472.CAN-14-0155

[5] Bray F , Ferlay J , Soerjomataram I , Siegel RL , Torre LA , Jemal A . Global cancer statistics 2018: GLOBOCAN estimates of incidence and mortality worldwide for 36 cancers in 185 countries. CA Cancer J Clin. 2018;68:394–424. 30207593 10.3322/caac.21492

[6] Ducreux M , Cuhna AS , Caramella C , et al. Cancer of the pancreas: ESMO Clinical Practice Guidelines for diagnosis, treatment and follow‐up. Ann Oncol. 2015;26(Suppl 5):v56–68. 26314780 10.1093/annonc/mdv295

[7] Conroy T , Desseigne F , Ychou M , et al. FOLFIRINOX versus gemcitabine for metastatic pancreatic cancer. N Engl J Med. 2011;364:1817–1825. 21561347 10.1056/NEJMoa1011923

[8] Von Hoff DD , Ervin T , Arena FP , et al. Increased survival in pancreatic cancer with nab‐paclitaxel plus gemcitabine. N Engl J Med. 2013;369:1691–1703. 24131140 10.1056/NEJMoa1304369 PMC4631139

[9] Yamaguchi K , Okusaka T , Shimizu K , et al. Clinical practice guidelines for pancreatic cancer 2016 from the Japan Pancreas Society: a synopsis. Pancreas. 2017;46:595–604. 28426492 10.1097/MPA.0000000000000816

[10] Nccn U . US NCCN Clinical Practice Guidelines in Oncology (NCCN Guidelines®). Pancreatic Adenocarcinoma Version 1.2019. 2018.

[11] Sohal DPS , Kennedy EB , Khorana A , et al. Metastatic pancreatic cancer: ASCO clinical practice guideline update. J Clin Oncol. 2018;36:2545–2556. 29791286 10.1200/JCO.2018.78.9636 PMC7504972

[12] Gill S , Ko Y‐J , Cripps C , et al. PANCREOX: a randomized phase III study of fluorouracil/leucovorin with or without oxaliplatin for second‐line advanced pancreatic cancer in patients who have received gemcitabine‐based chemotherapy. J Clin Oncol. 2016;34:3914–3920. 27621395 10.1200/JCO.2016.68.5776

[13] Oettle H , Riess H , Stieler JM , et al. Second‐line oxaliplatin, folinic acid, and fluorouracil versus folinic acid and fluorouracil alone for gemcitabine‐refractory pancreatic cancer: outcomes from the CONKO‐003 trial. J Clin Oncol. 2014;32:2423–2429. 24982456 10.1200/JCO.2013.53.6995

[14] Wang‐Gillam A , Hubner RA , Siveke JT , et al. NAPOLI‐1 phase 3 study of liposomal irinotecan in metastatic pancreatic cancer: final overall survival analysis and characteristics of long‐term survivors. Eur J Cancer. 2019;108:78–87. 30654298 10.1016/j.ejca.2018.12.007

[15] Ioka T , Komatsu Y , Mizuno N , et al. Randomised phase II trial of irinotecan plus S‐1 in patients with gemcitabine‐refractory pancreatic cancer. Br J Cancer. 2017;116:464–471. 28081543 10.1038/bjc.2016.436 PMC5318973

[16] Ioka T , Ueno M , Ueno H , et al. TAS‐118 (S‐1 plus leucovorin) versus S‐1 in patients with gemcitabine‐refractory advanced pancreatic cancer: a randomised, open‐label, phase 3 study (GRAPE trial). Eur J Cancer. 2019;106:78–88. 30471651 10.1016/j.ejca.2018.10.004

[17] Ohkawa S , Okusaka T , Isayama H , et al. Randomised phase II trial of S‐1 plus oxaliplatin vs S‐1 in patients with gemcitabine‐refractory pancreatic cancer. Br J Cancer. 2015;112:1428–1434. 25880004 10.1038/bjc.2015.103 PMC4453667

[18] Ueno M , Okusaka T , Omuro Y , et al. A randomized phase II study of S‐1 plus oral leucovorin versus S‐1 monotherapy in patients with gemcitabine‐refractory advanced pancreatic cancer. Ann Oncol. 2016;27:502–508. 26681680 10.1093/annonc/mdv603 PMC4769993

[19] Chiang N‐J , Chao T‐Y , Hsieh R‐K , et al. A phase I dose‐escalation study of PEP02 (irinotecan liposome injection) in combination with 5‐fluorouracil and leucovorin in advanced solid tumors. BMC Cancer. 2016;16:907. 27871319 10.1186/s12885-016-2933-6 PMC5117585

[20] Drummond DC , Noble CO , Guo Z , Hong K , Park JW , Kirpotin DB . Development of a highly active nanoliposomal irinotecan using a novel intraliposomal stabilization strategy. Cancer Res. 2006;66:3271–3277. 16540680 10.1158/0008-5472.CAN-05-4007

[21] Kalra AV , Kim J , Klinz SG , et al. Preclinical activity of nanoliposomal irinotecan is governed by tumor deposition and intratumor prodrug conversion. Cancer Res. 2014;74:7003–7013. 25273092 10.1158/0008-5472.CAN-14-0572

[22] Kawato Y , Aonuma M , Hirota Y , Kuga H , Sato K . Intracellular roles of SN‐38, a metabolite of the camptothecin derivative CPT‐11, in the antitumor effect of CPT‐11. Cancer Res. 1991;51:4187–4191. 1651156

[23] Roy AC , Park SR , Cunningham D , et al. A randomized phase II study of PEP02 (MM‐398), irinotecan or docetaxel as a second‐line therapy in patients with locally advanced or metastatic gastric or gastro‐oesophageal junction adenocarcinoma. Ann Oncol. 2013;24:1567–1573. 23406728 10.1093/annonc/mdt002

[24] Chen L‐T , Siveke JT , Wang‐Gillam A , et al. Survival with nal‐IRI (liposomal irinotecan) plus 5‐fluorouracil and leucovorin versus 5‐fluorouracil and leucovorin in per‐protocol and non‐per‐protocol populations of NAPOLI‐1: Expanded analysis of a global phase 3 trial. Eur J Cancer. 2018;105:71–78. 30414528 10.1016/j.ejca.2018.09.010

[25] Hubner RA , Cubillo A , Blanc J‐F , et al. Quality of life in metastatic pancreatic cancer patients receiving liposomal irinotecan plus 5‐fluorouracil and leucovorin. Eur J Cancer. 2019;106:24–33. 30458340 10.1016/j.ejca.2018.09.029

[26] Pelzer U , Blanc J‐F , Melisi D , et al. Quality‐adjusted survival with combination nal‐IRI+5‐FU/LV vs 5‐FU/LV alone in metastatic pancreatic cancer patients previously treated with gemcitabine‐based therapy: a Q‐TWiST analysis. Br J Cancer. 2017;116:1247–1253. 28350787 10.1038/bjc.2017.67 PMC5482729

[27] Wang‐Gillam A , Li C‐P , Bodoky G , et al. Nanoliposomal irinotecan with fluorouracil and folinic acid in metastatic pancreatic cancer after previous gemcitabine‐based therapy (NAPOLI‐1): a global, randomised, open‐label, phase 3 trial. Lancet. 2016;387:545–557. 26615328 10.1016/S0140-6736(15)00986-1

[28] ESMO Guidelines Committee . Appendix 6: Cancer of the pancreas: MCBS eUpdate published online 20 June 2017 (www.esmo.org/Guidelines/Gastrointestinal‐Cancers). Ann Oncol. 2017;28:iv157. 28881927 10.1093/annonc/mdx244

[29] Ioka T , Nakamori S , Sugimori K , et al. 132P ‐ Liposomal irinotecan (nal‐IRI) plus 5‐fluorouracil/levoleucovorin (5 FU/LV) vs 5‐FU/LV in Japanese patients (pts) with gemcitabine‐refractory metastatic pancreatic cancer (mPAC). Ann Oncol. 2019;30:ix47‐ix8.

[30] Pokataev I , Fedyanin M , Polyanskaya E , et al. Efficacy of platinum‐based chemotherapy and prognosis of patients with pancreatic cancer with homologous recombination deficiency: comparative analysis of published clinical studies. ESMO Open. 2020;5:e000578. 10.1136/esmoopen-2019-000578 PMC7003386 33551067

[31] Macarulla Mercadé T , Chen L‐T , Li C‐P , et al. Liposomal irinotecan +5‐FU/LV in metastatic pancreatic cancer: subgroup analyses of patient, tumor, and previous treatment characteristics in the pivotal NAPOLI‐1 trial. Pancreas. 2020;49:62–75. 31856081 10.1097/MPA.0000000000001455 PMC6946097

[32] Ko AH , Tempero MA , Shan Y‐S , et al. A multinational phase 2 study of nanoliposomal irinotecan sucrosofate (PEP02, MM‐398) for patients with gemcitabine‐refractory metastatic pancreatic cancer. Br J Cancer. 2013;109:920–925. 23880820 10.1038/bjc.2013.408 PMC3749576

