# Supplementary figures and images for: nal‐IRI+5‐FU/LV versus 5‐FU/LV in post‐gemcitabine metastatic pancreatic cancer: Randomized phase 2 trial in Japanese patients
Source: Cancer Med. 2020 Oct 25;9(24):9396–408. doi: 10.1002/cam4.3558 (PMC7774735; doi:10.1002/cam4.3558)

# Supplementary Figure 1

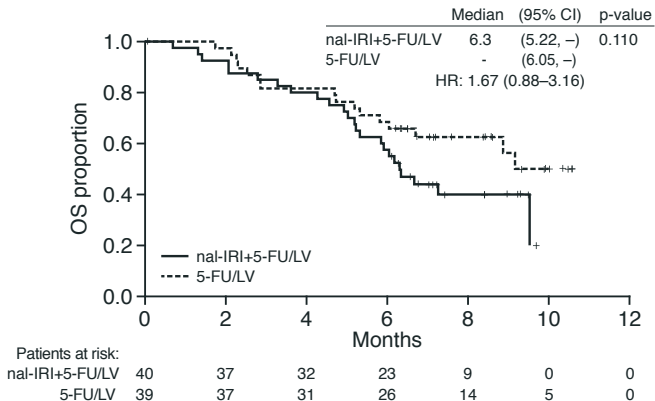

Supplement: Supplementary file 1 — Fig S1 [file CAM4-9-9396-s001.pdf]
