# Supplementary material for: nal‐IRI+5‐FU/LV versus 5‐FU/LV in post‐gemcitabine metastatic pancreatic cancer: Randomized phase 2 trial in Japanese patients
Source: Cancer Med. 2020 Oct 25;9(24):9396–408. doi: 10.1002/cam4.3558 (PMC7774735; doi:10.1002/cam4.3558)
Supplement: Supplementary file 2 — Table S1 [file CAM4-9-9396-s002.docx]

## Supplementary Table S1: Sensitivity analysis – Overall survival in Part 2 after censoring for front-line therapy (ITT population)

|  | **nal-IRI+5-FU/LV (n=40)** | **5-FU/LV  (n=39)** |
| --- | --- | --- |
| **Number of events (death), n (%)** | 18 (45.0) | 9 (23.1) |
| **Number of censors, n (%)** | 22 (55.0) | 30 (76.9) |
| **Reason for censoring, n (%)**  Censored at post-treatment front-line* start date Alive on cut-off date (May 4, 2017) Discontinued study | 10 (25.0) 12 (30.0) 0 | 23 (59.0) 6 (15.4) 1 (2.6) |
| **Median overall survival (months) (95% CI)** | 6.18 (5.03–NE) | 6.70 (2.86–NE) |
| p-value** HR (95% CI) | 0.805 1.11 (0.49–2.49) | |
| *Front-line regimen included gemcitabine-based combination regimens or FOLFIRINOX. **Two-sided p-value from log-rank test.  5-FU, 5-fluorouracil; CI, confidence interval, FOLFIRINOX, folinic acid, fluorouracil, irinotecan, oxaliplatin; HR, hazard ratio; ITT, intention-to-treat; LV, leucovorin; nal-IRI, liposomal irinotecan; NE, not evaluable. | | |
