# Supplementary material for: nal‐IRI+5‐FU/LV versus 5‐FU/LV in post‐gemcitabine metastatic pancreatic cancer: Randomized phase 2 trial in Japanese patients
Source: Cancer Med. 2020 Oct 25;9(24):9396–408. doi: 10.1002/cam4.3558 (PMC7774735; doi:10.1002/cam4.3558)
Supplement: Supplementary file 3 — Table S2 [file CAM4-9-9396-s003.docx]

## Supplementary Table S2**:** Summary of change from baseline for EORTC QLQ-C30 questionnaire by scales in Part 2 (PRO-evaluable population)

| **Time point** | **Parameter** | **nal-IRI+5-FU/LV**  **n=37** | | **5-FU/LV**  **n=36** | |
| --- | --- | --- | --- | --- | --- |
|  |  | **Observed** | **Change**  **from**  **baseline** | **Observed** | **Change**  **from**  **baseline** |
| **Global health status** | | | | | |
| Baseline | n | 37 | - | 36 | - |
|  | Median  (min, max) | 75.0  (8, 100) | - | 58.3  (33, 100) | - |
| Week 6 | n | 17 | 17 | 19 | 19 |
|  | Median  (min, max) | 66.7  (17, 100) | 0.0  (-50, 50) | 66.7  (25, 100) | 0.0  (-25, 33) |
| Week 12 | n | 13 | 13 | 5 | 5 |
|  | Median  (min, max) | 66.7  (25, 100) | 0.0  (-25, 50) | 83.3  (42, 92) | 8.3  (-42, 42) |
| **Physical functioning scale** | | | | | |
| Baseline | n | 37 | - | 36 | - |
|  | Median  (min, max) | 86.7  (40, 100) | - | 86.7  (47, 100) | - |
| Week 6 | n | 17 | 17 | 19 | 19 |
|  | Median  (min, max) | 93.3  (60, 100) | 0.0  (-27, 27) | 80.0  (13, 100) | -6.7  (-33, 27) |
| Week 12 | n | 13 | 13 | 5 | 5 |
|  | Median  (min, max) | 86.7  (53, 100) | 0.0  (-13, 40) | 86.7  (80, 100) | 0.0  (-7, 27) |
| **Role functioning scale** | | | | | |
| Baseline | n | 37 | - | 36 | - |
|  | Median  (min, max) | 83.3  (0, 100) | - | 83.3  (17, 100) | - |
| Week 6 | n | 17 | 17 | 19 | 19 |
|  | Median  (min, max) | 100.0  (33, 100) | 0.0  (-33, 50) | 66.7  (0, 100) | -16.7  (-50, 33) |
| Week 12 | n | 13 | 13 | 5 | 5 |
|  | Median  (min, max) | 83.3  (33, 100) | 0.0  (-33, 67) | 100.0  (67, 100) | 33.3  (0, 50) |
| **Cognitive functioning scale** | | | | | |
| Baseline | n | 37 | - | 36 | - |
|  | Median  (min, max) | 100.0  (0, 100) | - | 83.3  (33, 100) | - |
| Week 6 | n | 17 | 17 | 19 | 19 |
|  | Median  (min, max) | 83.3  (50, 100) | 0.0  (-33, 17) | 83.3  (17, 100) | 0.0  (-33, 33) |
| Week 12 | n | 13 | 13 | 5 | 5 |
|  | Median  (min, max) | 83.3  (50, 100) | 0.0  (-33, 33) | 83.3  (67, 100) | 0.0  (0, 33) |
| **Emotional functioning scale** | | | | | |
| Baseline | n | 37 | - | 36 | - |
|  | Median  (min, max) | 83.3  (42, 100) | - | 91.7  (58, 100) | - |
| Week 6 | n | 17 | 17 | 19 | 19 |
|  | Median  (min, max) | 91.7  (58, 100) | 0.0  (-17, 17) | 91.7  (0, 100) | 0.0  (-58, 17) |
| Week 12 | n | 13 | 13 | 5 | 5 |
|  | Median  (min, max) | 100.0  (58, 100) | 0.0  (-25, 42) | 91.7  (83, 100) | 0.0  (-8, 8) |
| **Social functioning scale** | | | | | |
| Baseline | n | 37 | - | 36 | - |
|  | Median  (min, max) | 100.0  (0, 100) | - | 83.3  (33, 100) | - |
| Week 6 | n | 17 | 17 | 19 | 19 |
|  | Median  (min, max) | 83.3  (33, 100) | 0.0  (-33, 33) | 83.3  (0, 100) | 0.0  (-83, 50) |
| Week 12 | n | 13 | 13 | 5 | 5 |
|  | Median  (min, max) | 100.0  (33, 100) | 0.0  (-33, 50) | 100.0  (83, 100) | 0.0  (0, 50) |
| **Fatigue symptom scale** | | | | | |
| Baseline | n | 37 | - | 36 | - |
|  | Median  (min, max) | 33.3  (0, 100) | - | 33.3  (0, 78) | - |
| Week 6 | n | 17 | 17 | 19 | 19 |
|  | Median  (min, max) | 33.3  (0, 56) | 0.0  (-22, 22) | 33.3  (0, 100) | 0.0  (-33, 33) |
| Week 12 | n | 13 | 13 | 5 | 5 |
|  | Median  (min, max) | 33.3  (0, 67) | 0.0  (-44, 11) | 22.2  (22, 33) | -11.1  (-22, 11) |
| **Pain symptom scale** | | | | | |
| Baseline | n | 37 | - | 36 | - |
|  | Median  (min, max) | 16.7  (0, 83) | - | 33.3  (0, 100) | - |
| Week 6 | n | 17 | 17 | 19 | 19 |
|  | Median  (min, max) | 16.7  (0, 33) | 0.0  (-33, 17) | 33.3  (0, 100) | 0.0  (-83, 33) |
| Week 12 | n | 13 | 13 | 5 | 5 |
|  | Median  (min, max) | 0.0  (0, 50) | 0.0  (-67, 33) | 16.7  (0, 33) | 0.0  (-67, 33) |
| **Nausea and vomiting symptom scale** | | | | | |
| Baseline | n | 37 | - | 36 | - |
|  | Median  (min, max) | 0.0  (0, 33) | - | 0.0  (0, 100) | - |
| Week 6 | n | 17 | 17 | 19 | 19 |
|  | Median  (min, max) | 0.0  (0, 33) | 0.0  (-33, 33) | 0.0  (0, 67) | 0.0  (-100, 50) |
| Week 12 | n | 13 | 13 | 5 | 5 |
|  | Median  (min, max) | 0.0  (0, 33) | 0.0  (-33, 17) | 0.0  (0, 0) | 0.0  (-33, 0) |
| **Dyspnea symptom scale** | | | | | |
| Baseline | n | 37 | - | 36 | - |
|  | Median  (min, max) | 0.0  (0, 67) | - | 0.0  (0, 67) | - |
| Week 6 | n | 17 | 17 | 19 | 19 |
|  | Median  (min, max) | 0.0  (0, 33) | 0.0  (-33, 33) | 0.0  (0, 67) | 0.0  (-67, 33) |
| Week 12 | n | 13 | 13 | 5 | 5 |
|  | Median  (min, max) | 33.3  (0, 33) | 0.0  (-33, 33) | 0.0  (0, 33) | 0.0  (-33, 0) |
| **Insomnia symptom scale** | | | | | |
| Baseline | n | 37 | - | 36 | - |
|  | Median  (min, max) | 0.0  (0, 100) | - | 0.0  (0, 100) | - |
| Week 6 | n | 17 | 17 | 19 | 19 |
|  | Median  (min, max) | 0.0  (0, 33) | 0.0  (-67, 0) | 0.0  (0, 100) | 0.0  (-67, 33) |
| Week 12 | n | 13 | 13 | 5 | 5 |
|  | Median  (min, max) | 0.0  (0, 67) | 0.0  (-67, 33) | 0.0  (0, 33) | 0.0  (-33, 33) |
| **Appetite loss symptom scale** | | | | | |
| Baseline | n | 37 | - | 36 | - |
|  | Median  (min, max) | 0.0  (0, 67) | - | 0.0  (0, 100) | - |
| Week 6 | n | 17 | 17 | 19 | 19 |
|  | Median  (min, max) | 0.0  (0, 67) | 0.0  (-33, 33) | 33.3  (0, 100) | 0.0  (-67, 67) |
| Week 12 | n | 13 | 13 | 5 | 5 |
|  | Median  (min, max) | 33.3  (0, 33) | 0.0  (-33, 33) | 0.0  (0, 33) | 0.0  (-33, 33) |
| **Constipation symptom scale** | | | | | |
| Baseline | n | 37 | - | 36 | - |
|  | Median (min, max) | 0.0  (0, 67) | - | 0.0  (0, 67) | - |
| Week 6 | n | 17 | 17 | 19 | 19 |
|  | Median (min, max) | 0.0  (0, 33) | 0.0  (-33, 33) | 0.0  (0, 100) | 0.0  (-33, 67) |
| Week 12 | n | 13 | 13 | 5 | 5 |
|  | Median (min, max) | 0.0  (0, 33) | 0.0  (-33, 33) | 0.0  (0, 33) | 0.0  (0, 33) |
| Diarrhea symptom scale | | | | | |
| Baseline | n | 37 | - | 36 | - |
|  | Median (min, max) | 0.0  (0, 67) | - | 0.0  (0, 67) | - |
| Week 6 | n | 17 | 17 | 19 | 19 |
|  | Median (min, max) | 0.0  (0, 67) | 0.0  (-67, 67) | 0.0  (0, 67) | 0.0  (-33, 33) |
| Week 12 | n | 13 | 13 | 5 | 5 |
|  | Median (min, max) | 33.3  (0, 33) | 0.0  (-67, 33) | 0.0  (0, 67) | 0.0  (0, 0) |
| Financial difficulties | | | | | |
| Baseline | n | 37 | - | 36 | - |
|  | Median (min, max) | 0.0  (0, 100) | - | 0.0  (0, 67) | - |
| Week 6 | n | 17 | 17 | 19 | 19 |
|  | Median (min, max) | 0.0  (0, 33) | 0.0  (-67, 33) | 0.0  (0, 100) | 0.0  (-33, 33) |
| Week 12 | n | 13 | 13 | 5 | 5 |
|  | Median (min, max) | 0.0  (0, 33) | 0.0  (-67, 33) | 0.0  (0, 0) | 0.0  (0, 0) |
| Week 6 corresponds to cycle 4 day 1, and week 12 to cycle 7 day 1.  5-FU, 5-fluorouracil; LV, leucovorin; nal-IRI, liposomal irinotecan; PRO, patient-reported outcome. | | | | | |
