# Supplementary material for: nal‐IRI+5‐FU/LV versus 5‐FU/LV in post‐gemcitabine metastatic pancreatic cancer: Randomized phase 2 trial in Japanese patients
Source: Cancer Med. 2020 Oct 25;9(24):9396–408. doi: 10.1002/cam4.3558 (PMC7774735; doi:10.1002/cam4.3558)
Supplement: Supplementary file 4 — Table S3 [file CAM4-9-9396-s004.docx]

## Supplementary table S3: TEAEs (any grade) resulting in dose delay, dose reduction (≥5% of patients in either treatment arm) or treatment discontinuation (≥2% of patients in either treatment arm) (safety population)

| **Parameter** | **nal-IRI+5-FU/LV**  **n=46** | **5-FU/LV**  **n=38** |
| --- | --- | --- |
| **Patients with TEAE leading to any dose modification,***  **n (%)** | 35 (76.1) | 12 (31.6) |
| **Dose delay**** | 32 (69.6) | 11 (28.9) |
| **Dose reduction^†^** | 23 (50.0) | 3 (7.9) |
| **Treatment discontinuation** | 10 (21.7) | 0 (0.0) |
| **Dose delay, n (%)** |  |  |
| White blood cell count decreased | 21 (45.7) | 2 (5.3) |
| Neutrophil count decreased | 20 (43.5) | 1 (2.6) |
| Diarrhea | 5 (10.9) | 0 |
| Neutropenia | 4 (8.7) | 0 |
| Constipation | 0 | 2 (5.3) |
| Platelet count decreased | 1 (2.2) | 2 (5.3) |
| **Dose reduction, n (%)** |  |  |
| Neutrophil count decreased | 11 (23.9) | 0 |
| Diarrhea | 8 (17.4) | 1 (2.6) |
| White blood cell count decreased | 7 (15.2) | 0 |
| Neutropenia | 3 (6.5) | 0 |
| **Treatment discontinuation, n (%)** | | |
| Pancreatic carcinoma | 2 (4.3) | 0 |
| Anemia | 1 (2.2) | 0 |
| Cerebral infarction | 1 (2.2) | 0 |
| Diarrhea | 1 (2.2) | 0 |
| Infection | 1 (2.2) | 0 |
| Myalgia | 1 (2.2) | 0 |
| Neutropenia | 1 (2.2) | 0 |
| Neutrophil count decreased | 1 (2.2) | 0 |
| Pleural effusion | 1 (2.2) | 0 |
| White blood cell count decreased | 1 (2.2) | 0 |
| **Patients with any TEAEs leading to death, n (%)** | 4 (8.7) | 2 (5.3) |
| Pancreatic carcinoma | 3 (6.5) | 2 (5.3) |
| Infection | 1 (2.2) | 0 |
| **TEAEs leading to death (all causes) related to study drug, n (%)** | 0 | 0 |
| The safety population comprised patients who received ≥1 dose of study drug during study Part 1 or 2.  TEAE was defined as an AE with an onset date or a pre-existing AE worsening following the first dose of study drug to 30 days after the last dose of study drug. AEs were coded according to MedDRA version 18.1.  *Dose modification: Dose reduction, dose interruption or dose withdrawn.  **Dose delay: Any action taken as infusion interrupted.  ^†^Dose reduction: Any action taken as dose decreased or slowing infusion rate.  5-FU, 5-fluorouracil; AE, adverse event; LV, leucovorin; nal-IRI, liposomal irinotecan; TEAE, treatment-emergent adverse event. | | |
