# Supplementary material for: nal‐IRI+5‐FU/LV versus 5‐FU/LV in post‐gemcitabine metastatic pancreatic cancer: Randomized phase 2 trial in Japanese patients
Source: Cancer Med. 2020 Oct 25;9(24):9396–408. doi: 10.1002/cam4.3558 (PMC7774735; doi:10.1002/cam4.3558)
Supplement: Supplementary file 5 [file CAM4-9-9396-s005.docx]

**Supplementary figure legend**

Figure S1 – Overall survival in study part 2 (ITT population). Kaplan-Meier plot of OS in the Part 2 ITT population. Tick marks indicate censoring points. 5-FU, 5‑fluorouracil; CI, confidence interval; HR, hazard ratio; ITT, intention-to-treat; LV, leucovorin; nal-IRI, liposomal irinotecan; OS, overall survival.
